# From infected to recovered: the mediating role of sleep quality between self-compassion, social support and COVID-19 psychosomatic symptoms

**DOI:** 10.1186/s12889-024-20657-9

**Published:** 2024-11-18

**Authors:** Outong Chen, Fang Guan, Chengqing Zhan, Ying Li

**Affiliations:** 1 Department of Psychology, Normal College & School of Teacher Education, Qingdao University, Qingdao, China; 2 School of Psychology, Third Military Medical University, Chongqing, China; 3 Fukang Hospital of Tibet University, Lhasa, China

**Keywords:** COVID-19 psychosomatic symptoms, Self-compassion, Sleep quality, Social support

## Abstract

**Background:**

Recent research has shown significant individual differences in COVID-19 psychosomatic symptoms. However, there has been a lack of studies investigating the influence of physical and psychological factors on these symptoms and their underlying mechanisms. This study aims to fill this gap by investigating the predictive role of self-compassion and social support on COVID-19 psychosomatic symptoms, as well as the potential mediating role of sleep quality.

**Methods:**

Data were collected from 636 participants infected with COVID-19 during the early post-pandemic reopening phase in China. The measurement tools used in the current study included the Self-Compassion Scale, the Perceived Social Support Scale, Self-Rating Scale of Sleep, and a COVID-19 Psychosomatic Symptom Diary.

**Results:**

A structural equation model revealed that: (1) social support directly predicts COVID-19 psychosomatic symptoms; (2) sleep quality fully mediates the relationship between self-compassion and COVID-19 psychosomatic symptoms; and (3) sleep quality partially mediates the relationship between social support and COVID-19 psychosomatic symptoms.

**Conclusions:**

These findings not only confirm previous research but also provide new insights into the intricate interplay between psychological and physical factors and their influence on COVID-19 psychosomatic symptoms. The implications of these findings may inform the development of targeted rehabilitation programs in the post-pandemic era of the “new normal”.

**Clinical trial number:**

Not applicable.

**Supplementary Information:**

The online version contains supplementary material available at 10.1186/s12889-024-20657-9.

## Introduction

The term “psychosomatic” refers to the interaction of psychological and biological factors with health and disease [1]. In the context of the COVID-19 pandemic, the psychosomatic symptoms associated with viral infection, such as insomnia, anxiety, depression, fatigue, pain and cognitive impairment, have received considerable research attention [2]. These symptoms require recovery periods to mitigate potential negative effects on physical and psychological health. Consequently, an increasing number of researchers are exploring the utilization of available resources, including internal psychological resources, to aid infected individuals in their recovery from the pandemic. Although the world has entered the so-called “COVID-19 post-pandemic era”, it is important to recognize that the crisis is not yet fully over. A previous study, based on 1, 002, 739 genomic sequences, revealed that the Coronavirus’ estimated genome-wide mutation rate is 1.0794 × 10^− 3^ per nucleotide per year [3]. This finding aligns with at least three predominant variants (Alpha, Delta, and Omicron) that have emerged since the initial outbreak in 2019. Prior studies have suggested that humanity may need to learn to coexist with this ongoing pandemic in our daily lives [4]. In light of this, considerable efforts have been dedicated to biological and medical research, aiming to develop new drugs such as Remdesivir, Molnupiravir, and Paxlovid to combat the COVID-19 pandemic [5]. Simultaneously, psychologists have focused on the role of psychological factors in facilitating recovery from COVID-19 psychosomatic symptoms at the individual level. Therefore, the current study aims to construct a latent parallel mediation model to elucidate the recovery processes involving physical factors (e.g., sleep quality), psychological factors (e.g., self-compassion and social support), and COVID-19 psychosomatic symptoms, as well as their underlying mechanisms.

### Self-compassion and COVID-19 psychosomatic symptoms

Based on a Buddhist perspective, self-compassion encompasses the way people relate to themselves in times of personal suffering, failure, and inadequacy [6]. It is rooted in the belief that others in the world also face similar or potentially even more challenging difficulties. Therefore, everyone, including oneself, deserves compassion [7]. The construct of self-compassion is conceptualized as a bipolar continuum ranging from compassionate self-responding to uncompassionate self-responding [8]. Specifically, Neff et al. [9] identify six elements of self-compassion: self-kindness, common humanity, mindfulness, self-judgment, isolation, and overidentification. Self-compassion is mainly regarded as a positive attitude and coping strategy toward life. In line with this concept, previous studies have demonstrated that higher levels of self-compassion are associated with better psychological health [10–12] and physical health [13–16]. For instance, Bellosta-Batalla et al. [17] conducted an 8-week compassion intervention with participants, and post-intervention questionnaires suggested a significant improvement in participants’ well-being. Moreover, measurements of immunoglobulin A levels showed a significant enhancement of participants’ immune function. More recently, a study conducted during the COVID-19 lockdown in Spain revealed that self-compassion plays a crucial role in coping with stress, anxiety, and depression [18, 19]. This positive effect was attributed to the emotion-regulating function of self-compassion [20]. It can therefore be hypothesized that self-compassion may help alleviate psychosomatic symptoms and enable a relatively rapid recovery from a COVID-19 infection. Based on previous research, the current study posits the first hypothesis:

#### Hypothesis 1

Higher self-compassion could predict milder COVID-19 psychosomatic symptoms among infected individuals.

### Social support and COVID-19 psychosomatic symptoms

Vaux [21] defines social support as the perception of being cared for by others and having access to tangible and intangible supportive resources. These supportive resources encompass emotional support, informational support, financial support, companionship, and more. Social support can stem from various sources, including spouses or companions, family members, friends, colleagues, and community connections [22]. Previous studies have consistently demonstrated the significant benefits of social support. According to the stress and coping theory of social support, it serves as a buffer against stress, mitigating the impact of stressful life events on health [23]. Schwarzer and Knoll [24] propose that social support acts as a resource that influences cognitive appraisal when encountering stressful events. As individuals face negative stimuli, the more support they receive, the better cognitive appraisal they produce, and the better their coping is facilitated. Consistently, Jakobsen et al. [25] conducted a study with 3681 adolescents, revealing a positive correlation between social support and multiple indicators of mental health, such as meaningfulness and subjective well-being. Furthermore, social support may contribute to individuals’ physical health. In a study by Czajkowski et al. [26], greater social support was identified to lower cardiovascular disease risk and improve disease outcomes. Additionally, insufficient social support was found to contribute to post-surgery frailty in patients [27]. During the COVID-19 pandemic, individuals with adequate social support demonstrated a reduced risk of health problems, including depression, anxiety, and sleep loss [28]. The adoption of self-isolation policies across nations aimed to limit the spread of COVID-19 infection. However, as social beings, people naturally seek connection with others to protect themselves from environmental factors [29]. These policies brought about uncertainty and concern, but social support has been shown to mitigate the impact on mental health [30]. In conclusion, the second hypothesis of the current study was as follows:

#### Hypothesis 2

Higher social support could predict milder COVID-19 psychosomatic symptoms among infected individuals.

### Sleep quality as a mediator

In various fields of sleep-related research, numerous metrics have been employed to measure individuals’ sleep. Among them, sleep quality refers to the subjective indices of how sleep is experienced including the feeling of being rested when waking up and satisfaction with sleep [31]. Spielman et al. [32] proposed a 3-P model that outlines how multiple factors can disrupt sleep quality and eventually lead to insomnia. These factors can be categorized as predisposing, precipitating, and perpetuating factors. Predisposing factors can be understood as biological factors, such as genes [33]. Precipitating factors refer to environmental factors, such as stress [34]. Perpetuating factors include behavioral and cognitive factors, such as sleep habits, and sleep-related anxiety [35].

Self-compassion, regarded as a perpetuating factor in the 3-P model, may play a significant role in elevate sleep quality. When considering sleep quality, higher levels of self-compassion can assist in addressing sleep disturbances, such as self-critical thoughts, leading to a reduction in restlessness and agitation, thereby promoting sleep quality [36]. A diary study conducted by Hu et al. [37] supported this reasoning and revealed that self-compassion increased individuals’ sleep quality. From a broader perspective, a meta-analysis examined the relationship between self-compassion and sleep quality. After including 15 publications, results suggested that higher self-compassion is associated with better sleep quality [38]. On the other hand, social support has been shown to affect sleep quality. According to the 3-P model, the precipitating factor covers many factors, such as stress [39], which can significantly impact sleep quality. In line with the transactional stress theory of social support proposed by Schwarzer and Knoll [24], social support is considered a valuable resource that influences the cognitive appraisal of stressful encounters. The more support is available, the better coping results are facilitated. Consequently, social support was an essential resource for effectively managing stress, ultimately facilitating better sleep quality. Supporting this notion, Grey et al. [28] found that higher levels of social support reduced the risk of depression and promoted better sleep quality during the COVID-19 pandemic. Thus, the current study hypothesizes that self-compassion and social support may positively predict sleep quality.

Moreover, sleep plays a crucial role in maintaining individuals’ physical and mental health [40, 41], as it affects both the sympathetic nervous system and the hypothalamic-pituitary-adrenal axis, which are integral to regulating both the innate and adaptive immune responses [42]. A study on sport-related concussion adolescents revealed that better sleep quality predicted lower symptom severity and shorter recovery time after concussion [43]. Therefore, it is reasonable to assume that better sleep quality could contribute to a faster recovery of patients infected with COVID-19 by supporting relatively better immune function. In conclusion, the third hypothesis of the current study can be formulated as follows:

#### Hypothesis 3

Sleep quality could mediate the relationship between self-compassion, social support, and COVID-19 psychosomatic symptoms.

Taken together, while self-compassion, social support, and sleep quality were theoretically associated with COVID-19 psychosomatic symptoms, empirical evidence is necessary to support these claims. Following the 3-P model of sleep quality by Spielman et al. [32], the current study constructed a parallel mediation model using the latent-variable approach to gain insights into the recovery processes after COVID-19 infection. Building upon the literature review, the study aims to investigate whether self-compassion and social support can independently predict COVID-19 psychosomatic symptoms, and whether sleep quality can mediate these relationships.

## Method

### Participant

953 participants were recruited to participate in the study program during the early COVID-19 reopening in China, from December 2022 to January 2023. This period witnessed a significant surge in infections within a short time frame. They responded to a series of measures through Credamo [44], a professional data collection and business application platform similar to Mturk. They were required to respond to questionnaires about personality and individual differences. After completing the questionnaires, all the participants were notified that they would continue to report COVID-19 psychosomatic symptoms if they were confirmed to be infected. After excluding the uninfected, asymptomatic infected, recovery time of less than 7 days patients, and cases with missing data, data from 636 participants (393 women and 243 men, *M*_age_ = 30.90, *SD*_age_ = 8.17, Table 1) were retained for further statistical analysis. All subjects gave written informed consent in accordance with the Declaration of Helsinki. Participants who completed the survey were thanked for their participation and received monetary compensation (¥15). Additionally, information about relevant counseling services was provided both at the beginning and end of the survey.

**Table 1:** Participants’ demographic data (*N* = 636)

		Participants in the analytic sample (*N* = 636)
Gender	Men	243
	Women	393
Age	Mean (SD)	30.93 (8.17)
Ethnicity	Yao, n (%)	1(0.2%)
	Dai, n (%)	1(0.2%)
	Dong, n (%)	1(0.2%)
	Yi, n (%)	1(0.2%)
	Manchu, n (%)	2(0.3%)
	Tujia, n (%)	2(0.3%)
	Zhuang, n (%)	5(0.8%)
	Hui, n (%)	6(0.9%)
	Han, n (%)	617(97%)
Religion	Buddhism	1(0.2%)
	Christian	27(4.2%)
	Taoism	59(9.3%)
	Islam	426(67%)
	None	123(19.3%)
Place of residence	Living alone	36(5.7%)
	Living with family	572(89.9%)
	Group apartment or dormitory	24(3.8%)
	Hospital	2(0.3%)
	Other	2(0.3%)
Cigarette usage	None	538(84.6%)
	<10 per day	78(12.3%)
	10~19 per day	18(82.8%)
	>20 per day	2(0.3%)
Alcohol usage	None	460(872.3%)
	≤3 per week(female); ≤6 per week(male)	168(26.4%)
	4~7 per week(female); 6~14 per week(male)	8(1.3%)
	≥8 per week(female); ≥15 per week(male)	
Personal exercise habits	Never	81(12.7%)
	Everyday	86(13.5%)
	1~2 per week	291(45.8%)
	≥3 per week	178(28%)
Body weight	lean	58(9.1%)
	Normal	447(70.3%)
	Fat	119(18.7%)
	Obesity	12(1.9%)
Vaccination status	1	2(0.3%)
	2	61(9.6%)
	3	554(87.1%)
	4	16(2.5%)
	None	2(0.3%)
	Missing value	1(0.2%)

### Measures

#### Self-compassion

Participants’ self-compassion levels were measured by a translated and revised version of the Self-Compassion Scale [7]. Participants were asked to respond to each item on a scale from 1 (*never*) to 5 (*all the time*), with higher scores representing higher self-compassion levels. This scale consists of 6 subscales, 26 items, and the sample item is as follows: *I’m disapproving and judgmental about my flaws and inadequacies.* The total score of the Self-compassion Scale served as the independent variable in the hypothetical model. The Cronbach’s α in this study was 0.93.

#### Social support

A revised and translated version of the Perceived Social Support Scale from Zimet et al. [45] was adopted to measure participants’ social support levels in the current study through self-report. They were asked to rate 12 items from 1 (*totally disagree*) to 7 (*totally agree*), with higher scores representing higher social support levels. An example item from the scale is: *There is a special person who is around when I am in need*. This scale showed adequate internal consistency in the current study (full-scale Cronbach’s α = 0.93).

#### Sleep quality

Sleep quality in the current study was evaluated by the Self-Rating Scale of Sleep developed by Li [46]. Participants were asked to respond to 10 items on a scale from 1 (*never*) to 5 (*always*). One sample item is: *Experience frequent dreams or is frequently awakened by nightmares during the last month?* The total score ranges from 10 to 50, which represents participants’ sleep quality. It is worth noting that after reverse scoring, the higher the total score is, the better the quality of sleep. The Cronbach’s α of this scale in the current study was 0.76.

#### COVID-19 psychosomatic symptoms

Based on the research of the World Health Organization [47] and multiple other research, such as Wiegele et al. [48], the current study adopted a translated COVID-19 Psychosomatic Symptom Diary to measure participants’ relative symptoms. Studies suggest that the predominant variant of COVID-19 during China’s re-opening outbreak is Omicron [49]. Moreover, research evidences have shown that individuals infected by Omicron are more likely to recover within a week [50]. Thus, participants were asked to retrospectively report a 7-days variation of their COVID-19 psychosomatic symptoms from the first day when they were infected. Following COVID-19 infection, individuals typically experience symptoms such as fever and headache, necessitating rest and limiting their ability to use devices like mobile phones and computers. Consequently, data collection relied on participants’ memory and self-reports. Although the participants were not currently experiencing symptoms while filling the questionnaires, they were generally at the end of their illness. Results were collected on a scale from 0 (*none*) to 7 (*severe*). The symptoms included: *Fever*, *Runny nose*, *Lost of taste &smell*, etc. Notably, the mediating variable of the current study was sleep quality. In order to avoid variable confusion, items in the symptom diary related to sleep were excluded in the current study. Considering that some of the symptoms in this study are also manifested in daily life, or are left over from a previous COVID infection, some researchers refer to this as “Long-COVID” [51, 52], the present study synchronized the collection of data on whether the participants exhibited certain symptoms prior to confirming their infection as day 0 data for inclusion in the statistical analysis. Symptoms scores were averaged to represent the overall severity of each symptom. Additionally, the total score of all symptoms was calculated as the dependent variable in the proposed model. The Cronbach’s α of the COVID-19 psychosomatic symptom diary in the current study was 0.92.

#### Covariates

To account for various factors that may influence COVID-19 outcomes, several demographic information was collected as covariates in the current study, following the suggestion of Zvolensky et al. [53]. The demographic information encompassed details such as ethnicity, place of residence, religion, education, marital status, alcohol and cigarette usage, inflammatory conditions, personal exercise habits, body weight, and vaccination status. Notably, the participants were informed that all the information asked to fill in referred to their daily status before COVID-19 infection.

### Procedure

All participants in the study answered the measures anonymously. They were informed that the data would only be collected for research purposes, and that their privacy would be well protected. They were also informed of their right to withdraw from the study at any time if they felt uncomfortable or no longer wished to participant. Participants were initially informed that they were recruited to participate in a survey about personality. Self-compassion, social support, sleep quality, COVID-19 psychosomatic symptom, and demographic information were collected. After completing the questionnaire, they were sincerely thanked for their participation, and received appropriate compensation as a token of appreciation for their time and effort.

### Common method bias

Although the questionnaires in the current study were collected anonymously and some items were reverse scored to avoid potential bias, the Harman single-factor method was adopted to indicate the common method bias in the current study [54]. The exploratory factor analysis obtained 6 factors in total. The interpretation rate of the first factor was 9.66%, which was below the recommended threshold (40%). The results indicate that no serious common method bias was detected in the current study.

## Data analysis

A two-step analysis strategy proposed by Anderson and Gerbing [55] was adopted: (1) conducting confirmatory factor analysis (CFA) on all measurements to establish an adequate measurement model; (2) evaluating the structural model considering a direct effect of self-compassion and social support on COVID-19 psychosomatic symptoms and indirect effects through sleep quality, among latent constructs. χ^2^/df, RMSEA (root-mean square error of approximation), SRMR (standardized root mean square residual), CFI (comparative fit index) and TLI (Tucker–Lewis index) were calculated to assess the acceptability of the SEM and CFA model. Finally, the direct and indirect effects were tested by bias-corrected bootstrap with 5000 random replicate samples. SPSS 26.0 was used for descriptive statistics and Mplus 8.3 [56] was used for the structural equation model (SEM) among latent variables using maximum likelihood estimation.

Item parceling was employed to represent the relationships between latent variables. The Social Support Scale and the Self-Compassion Scale were designed as multidimensional scale, while the Sleep Quality Scale and the COVID-19 Psychosomatic Symptom Diary were unidimensional scales. Referring to Wu and Wen [57], the item parcels of sleep quality and COVID-19 psychosomatic symptoms were created according to the factorial algorithm (item-to-construct balance). The item parcels of self-compassion and social support were created using an internal-consistency approach, while the total score of each subscale representing the corresponding item parcels. The hypothesized model comprised 4 latent and 16 observed variables: 6-item parcels were created for the self-compassion factor, 3-item parcels were created for the social support factor, 3-item parcels were created for the sleep quality factor, and 4-item parcels were created for the COVID-19 psychosomatic symptoms factor. Additionally, some demographic information was collected during the survey, they were controlled as covariates in the analysis.

## Results

### Descriptive analysis

**Fig. 1:**
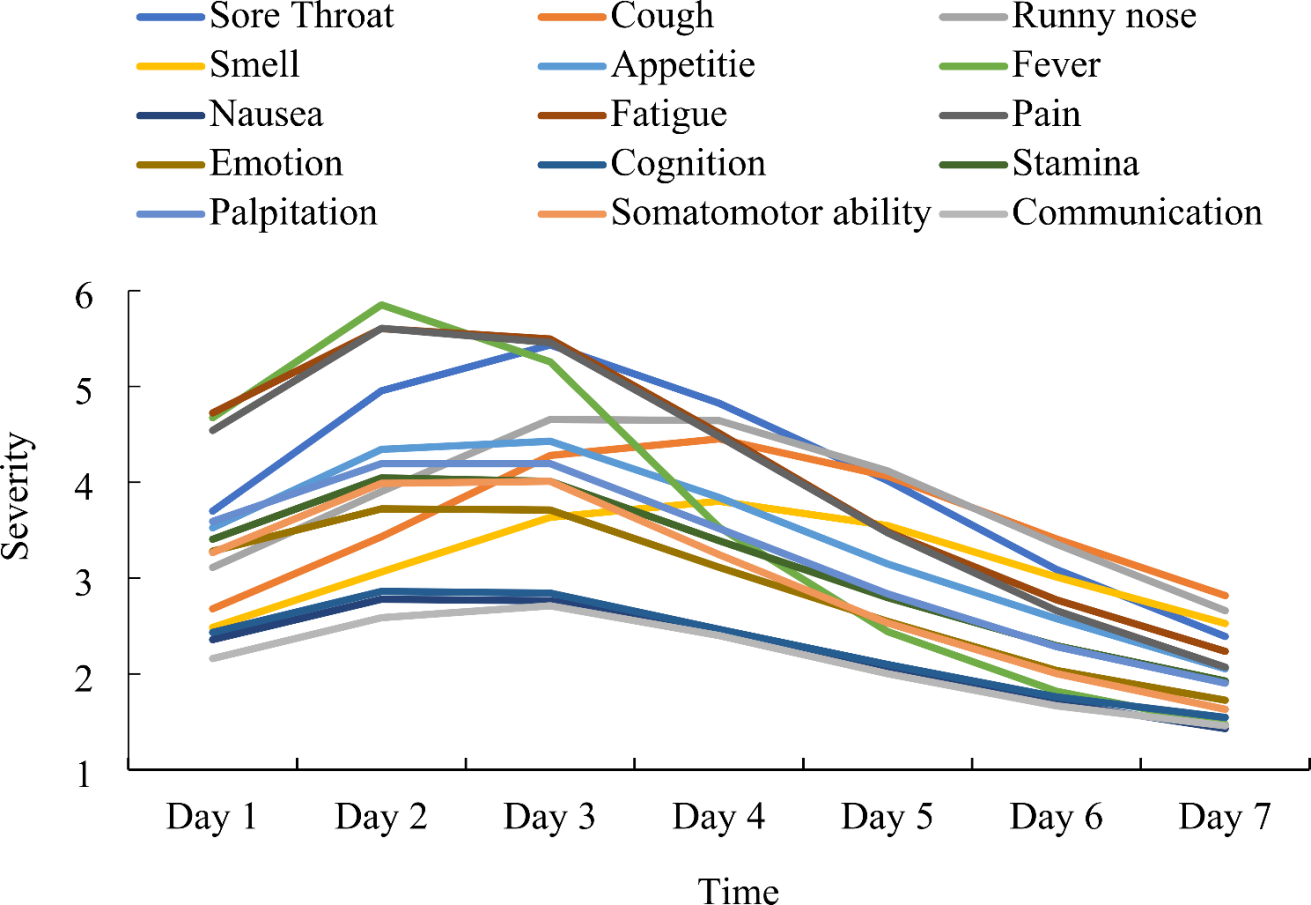
COVID-19 psychosomatic symptoms change over time. Note: Symptoms were measured on a scale from 0 (none) to 7 (severe). The ordinate shows the average score of 636 participants

Figure 1 demonstrate descriptive information regarding each symptom across seven days.

After averaging all the symptoms over seven days, correlations were calculated with other research variables. The final score for each scale was calculated by averaging the scores of all items within that scale. Table 2 shows the means, standard deviations, and correlation coefficients between every examined variable. Self-compassion was positively correlated with social support (*r* = .60, *p* < .01, 95% CI [0.55, 0.65]) and sleep quality (*r* = .49, *p* < .01, 95% CI [0.44, 0.55]). Additionally, it also negatively correlated with COVID-19 psychosomatic symptoms (*r* = -.12, *p* < .01, 95% CI [-0.19, -0.04]). Social support was positively correlated with sleep quality (*r* = .39, *p* < .01, 95% CI [0.32, 0.45]). Furthermore, sleep quality was negatively correlated with COVID-19 psychosomatic symptoms (*r* = -.18, *p* < .01, 95% CI [-0.25, -0.10]). Notably, demographic information was collected during our survey, including age, gender, residency status, use of cigarettes and alcohol, marital status, and most importantly, COVID-19 vaccine uptake (Table 1). However, they only yielded weak or non-significant relations with the main studied variable. The correlation coefficients of each covariate were not reported in this section, but they will be controlled as covariates in the current mediation model.

**Table 2:** Means, standard deviations, and correlations of the variables

Variables	M(SD)	1	2	3	4
1. Self-compassion	3.67(0.59)	-			
2. Social Support	5.70(0.82)	0.60^**^	-		
3. Sleep Quality	1.89(0.48)	0.49^**^	0.39^**^	-	
4.COVID-19 psychosomatic symptoms	2.97(0.94)	-0.12^**^	-0.03	-0.18^**^	-

Note *M* = Mean; *SD* = Standard Deviation; ^*^p < .05, ^**^p < .01

### Testing for mediated association

#### Measurement model

**Fig. 2:**
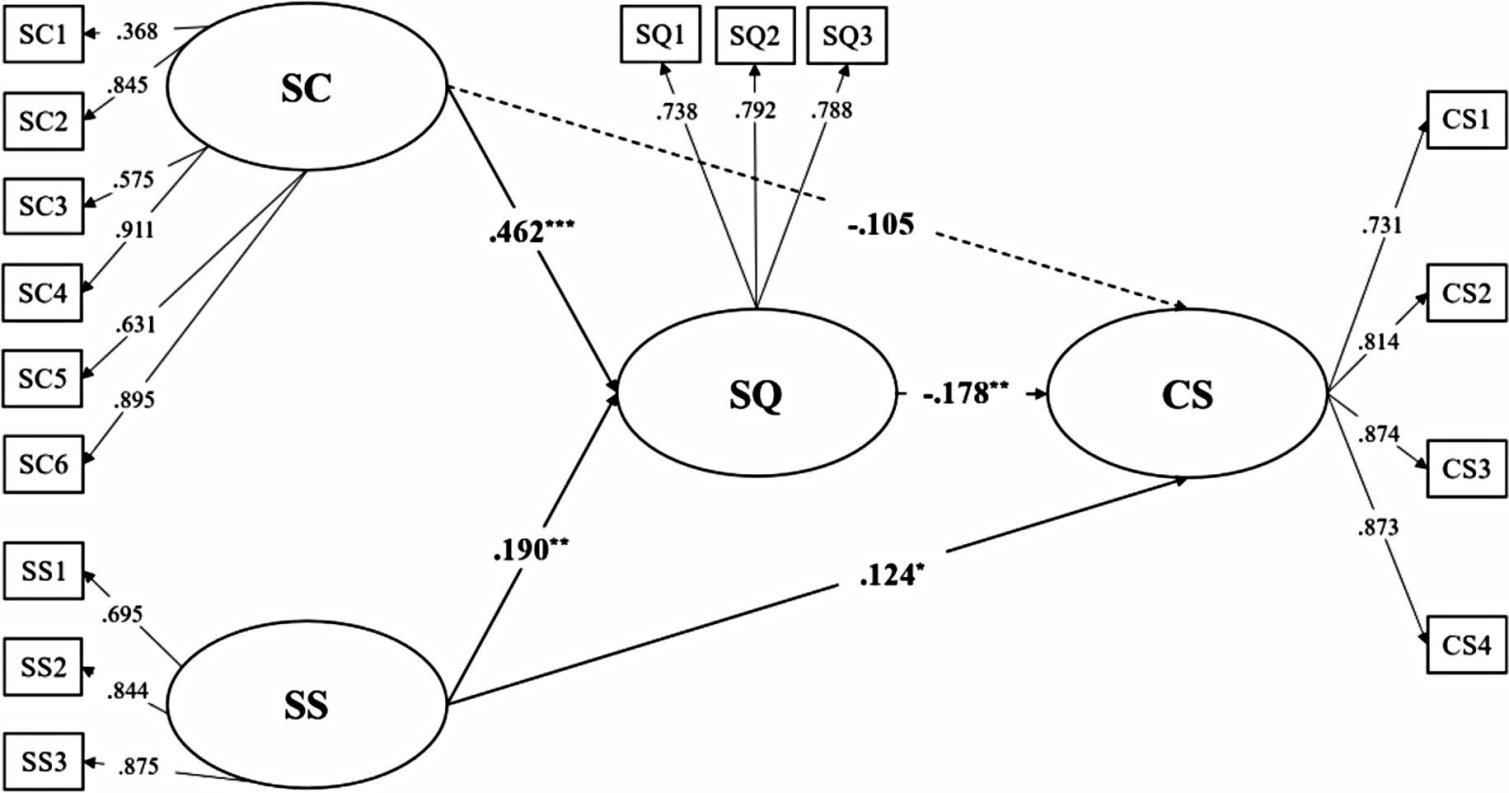
Mediating role of sleep quality between self-compassion, social support, and COVID-19 psychosomatic symptoms (*N* = 636). SC = self-compassion, SS = social support, SQ = sleep quality, CS = COVID-19 psychosomatic symptoms; All the factor loadings are standardized. SC1-SC6 are six parcels of self-compassion; SS1-SS3 are three parcels of social support; SQ1-SQ3 are three parcels of sleep quality; CS1-CS4 are 4 parcels of COVID-19 psychosomatic symptoms. ^*^p < .05, ^**^p < .01, ^***^p < .001

The current model consists of four latent variables and 16 observed variables. Results of a confirmatory factor analysis revealed that the goodness-of-fit for the measurement model was satisfactory: *χ*^*2*^*/df* = 2.89, RMSEA = 0.06, CFI = 0.97, TLI = 0.96, SRMR = 0.06. This result suggests that all the indicators well represent their latent factor respectively. Furthermore, all factor loadings for the indicators on the latent factors were significant (Fig. 2).

#### Structural model

Firstly, the proposed model fitted well with the data: *χ*^*2*^*/df* = 3.91, RMSEA = 0.07, CFI = 0.91, TLI = 0.90, SRMR = 0.08. Secondly, to further reveal the indirect effects of sleep quality between self-compassion, social support, and COVID-19 psychosomatic symptoms (see Fig. 2), the bootstrap (*n* = 5000) procedure was used to calculate the CI for all possible indirect effects. Specifically, the indirect effect of self-compassion on COVID-19 psychosomatic symptoms mediated by sleep quality was estimated at -0.08 (95% CI: -0.15 ~ -0.02), as illustrated in Table 3. Moreover, the indirect effect of social support on COVID-19 psychosomatic symptoms mediated by sleep quality was also significant (Estimated indirect effect: -0.03, 95% CI: -0.08 ~ -0.01). Notably, the direct effect of self-compassion on COVID-19 psychosomatic symptoms was not significant (Direct effect = -0.11, *p* = .13, 95% CI: -0.25 ~ -0.03) whereas the direct effect of social support on COVID-19 psychosomatic symptoms was significant (Direct effect = 0.13, *p* < .05, 95% CI: 0.004 ~ 0.253). This result indicated that sleep quality totally mediated the relationship between self-compassion and COVID-19. However, it only partially mediated the relationship between social support and COVID-19 psychosomatic symptoms.

**Table 3:** Standardized indirect effects and their 95% confident intervals

Model path	Total effect	Direct effect	Indirect effect	95% CI of Indirect effect	
				Lower	Upper
SC→SQ→CS	-0.19^**^	-0.11	-0.08^*^	-0.15	-0.02
SS→SQ→CS	0.09	0.13^*^	-0.03^**†**^	-0.08	-0.01

Note SC = self-compassion, SS = social support, SQ = sleep quality, CS = COVID-19 psychosomatic symptoms; 95% CI = 95% confidence intervals; ^†^*p* < .10, ^*^p < .05, ^**^p < .01

## Discussion

The current study investigated the relationship between self-compassion, social support, sleep quality, and COVID-19 psychosomatic symptoms in a sample of people in China infected with COVID-19. The results indicated that higher levels of self-compassion and social support were associated with better sleep quality, which, in turn, predicted lower severity of COVID-19 psychosomatic symptoms (hypothesis 3). Specifically, sleep quality totally mediated the relationship between self-compassion and COVID-19 psychosomatic symptoms. It also partially mediated the relationship between social support and COVID-19 psychosomatic symptoms. In addition, the study also yielded unexpected results. Hypothesis 1 of the current study was not supported, as the direct effect between self-compassion and COVID-19 psychosomatic symptoms was not significant. Similarly, hypothesis 2 was also not validated, with the predictive effect of social support on COVID-19 psychosomatic symptoms being positive.

Firstly, sleep quality negatively predicted COVID-19 psychosomatic symptoms individually (hypothesis 3). Sleep plays a vital role in maintaining individuals’ both physical and mental health. It is important to maintain daily sleep quality, especially during COVID-19 infection. Adaptive immunity and innate immunity constitute the human immune system. Wherein, the adaptive immune system responds to infectious challenges. The antigen-presenting cells are attracted to a site of intrusion, such as the coronavirus, take up invading antigen, and then migrate to local lymph nodes [58, 59]. Furthermore, such immune function is mainly regulated by sleep [60]. During nocturnal sleep, the function of immune cells, such as the T cell, is enhanced [61]. Furthermore, even a modest amount of sleep has been found to increase the production of interleukin [62], which is important for immune function. The positive association between sleep quality and patients’ recovery, as observed in the study by Ibarra-Coronado et al. and Silva et al. [43, 63], is consistent with the current findings. This suggests that better sleep quality is linked to a reduced severity of COVID-19 psychosomatic symptoms. These findings align with previous research emphasizing the importance of sleep in maintaining optimal immune function and overall health.

Importantly, the current study revealed the mediating role of sleep quality between self-compassion, social support and COVID-19 psychosomatic symptoms (hypothesis 3). Specifically, sleep quality totally mediated the predictive effect between self-compassion and COVID-19 psychosomatic symptoms while partially mediating the predictive effect between social support and COVID-19 psychosomatic symptoms. Although there are evidences suggest that self-compassion may facilitate individuals’ immune system [17], the direct effect of self-compassion on COVID-19 psychosomatic symptoms in the current study was not significant. The current results indicate that the only pathway through which self-compassion affects COVID-19 psychosomatic symptoms is via sleep quality. This is consistent with previous research [37, 64]. This suggests that the effects of self-compassion are more pronounced on the psychological level rather than the physiological level. The role of self-compassion in regulating negative emotions can be effectively integrated into the 3-P model of sleep proposed by Spielman et al. [32].

In addition, sleep quality also partially mediated the predictive effect of social support on COVID-19 psychosomatic symptoms. The direct effect of social support is discussed below. As for the indirect effect, existing research evidence indicates that social support serves as an effective resource for individuals to cope with negative psychological factors (such as emotions, cognition, and behavior) encountered in daily life [24, 25]. Within the context of this study, social support effectively buffers the negative cognitions and emotions induced by the COVID-19 pandemic, such as anxiety and stress, which have been demonstrated in previous research to adversely affect sleep [65]. Consequently, better social support exerts a protective effect on individuals’ sleep quality. Furthermore, when individuals’ sleep is adequately ensured, psychological resources can be better restored [66]. Additionally, good sleep quality ensures the proper functioning of the immune system [43, 63]. Therefore, sleep quality mediates the relationship between social support and symptoms.

Interestingly, specific findings of the current study diverged from our initial hypotheses. Firstly, the direct effect of self-compassion on COVID-19 psychosomatic symptoms was not significant. This finding suggests that the primary role of self-compassion lies in enhancing sleep quality, which indirectly aids in mitigating and recovering from COVID-19 symptoms. No other independent effects of self-compassion were identified in this study. Secondly, a significant direct effect of social support on COVID-19 psychosomatic symptoms was revealed, which is contrary to our expectation. However, these results can be better understood in light of previous research. As for self-compassion (hypothesis 1), Neff [16] suggested that self-compassion on physical health is mediated by its direct influence on health-promoting behaviors. In other words, self-compassion indirectly enhances physical health by promoting behaviors such as reduced tobacco and alcohol use, a healthy diet, regular exercise, seeking medical care, and importantly, maintaining good sleep habits [67]. These insights are consistent with the present finding, where sleep fully mediated the relationship between self-compassion and COVID-19 psychosomatic symptoms. Previous research has shown that the individual and societal impacts of COVID-19 can persist for several years, manifesting as widespread anxiety and depression [52]. The critical psychological function of self-compassion may serve as a valuable tool in mitigating the long-term negative psychological effects induced by COVID-19.

Contrary to our hypothesis 2, social support and COVID-19 psychosomatic symptoms were unexpectedly positively correlated in the current study. After reviewing multiple relative studies, the current study hypothesized that social support could negatively predict COVID-19 psychosomatic symptoms. However, the results unveiled a complex relationship, suggesting a suppressing effect, as described by MacKinnon [68], between social support, sleep quality, and COVID-19 psychosomatic symptoms. Specifically, social support exhibited a direct positive effect on COVID-19 psychosomatic symptoms, but it also had a negative indirect effect on COVID-19 psychosomatic symptoms through its prediction on sleep quality. One possible explanation for these unexpected findings was the Signaling Theory of Symptoms proposed by Steinkopf [69]. According to this theory, external representation of disease symptoms, such as fever, swelling, noticeable signs of pain, serve not only as defensive and healing mechanisms but also as signals to attract attention and support from potential helpers. In the context of the current study, individuals infected with COVID-19 may exaggerate the amount and severity of their symptoms to heighten the likelihood of mobilizing help and attention from close contacts. Therefore, the positive predictive effect of social support on COVID-19 psychosomatic symptoms could be interpreted within the framework of the Signaling Theory of Symptoms. This discrepancy highlights the need to continue to explore the reliability of these unexpected results as well as other potential explanations in future studies.

It is essential to acknowledge the limitations of the current study. Firstly, the cross-section design employed in this research prevented us from revealing causal relationships between the variables under investigation. Future research needs to overcome the challenges associated with longitudinal and experimental studies. A longitudinal design requires measuring independent/mediating variables at one time point and then measuring the dependent variables with a delay of 6 to 12 months. However, in the current study, the dependent variable is the symptoms of COVID-19 infection, making it nearly impossible to predict whether a participant will be infected at a specific moment. Additionally, according to Menni et al. [50], recovery from such an infection typically occurs within seven days. Moreover, our study was conducted during a time when China’s national COVID-19 policies had been completely lifted, making future social contexts and policy changes unpredictable factors. Secondly, the current study mainly relied on self-reported measures to assess the severity of participants’ COVID-19 psychosomatic symptoms. While self-report measures are valuable, incorporating additional objective indicators, such as blood tests or medical records, would enhance the robustness and reliability of the findings. Future studies should consider including a more comprehensive range of assessment tools. Thirdly, the positive relationship observed between social support and COVID-19 psychosomatic symptoms, as interpreted by the Signaling Theory of Symptoms, warrants further exploration and a more detailed examination. Additional research is needed to delve into the underlying mechanisms and provide a more comprehensive understanding of this relationship.

Notwithstanding these limitations, the present study provides valuable insights into the interplay among self-compassion, social support, sleep quality and COVID-19 psychosomatic symptoms. Notably, sleep quality was found to play a mediating role in the associations between self-compassion, social support, and COVID-19 psychosomatic symptoms. Furthermore, social support exhibited a positive individual predictive effect on COVID-19 psychosomatic symptoms while also exerting a negative indirect effect through its impact on sleep quality, which resulted in a non-significant total effect. These findings contribute to our understanding of the multifaceted nature of these variables and underscore the importance of considering sleep quality as a mediator in the relationship between self-compassion, social support, and COVID-19 psychosomatic symptoms, providing valuable insights and setting the stage for further exploration and refinement of these relationships in future research.

## Electronic supplementary material

Below is the link to the electronic supplementary material.

Supplementary Material 1

## Data Availability

The data that support the findings of this study are available from the corresponding author upon reasonable request.
