# Supplementary material for: From infected to recovered: the mediating role of sleep quality between self-compassion, social support and COVID-19 psychosomatic symptoms
Source: BMC Public Health. 2024 Nov 18;24:3196. doi: 10.1186/s12889-024-20657-9 (PMC11571965; doi:10.1186/s12889-024-20657-9)
Supplement: Supplementary file 1 — Supplementary Material 1 [file 12889_2024_20657_MOESM1_ESM.docx]

## Additional File

*This file includes the instructions, scoring methods, and reference sources for the questionnaires\scales used in this study. Both the original items and the translated and revised items were included.*

# 新冠症状日记（COVID-19 Psychosomatic Symptom Diary）

请您在下面的表格中填写自己会出现的症状及严重程度，并给每个症状可能的严重程度打分。

Please fill in the symptoms and severity in the form below and rate the possible severity of each symptom.

0= None /几乎没有 7= Severe /非常严重

|  | 第一天  Day 1 | 第二天  Day 2 | ……. | 第七天  Day 7 |
| --- | --- | --- | --- | --- |
| 1.喉咙敏感/声音变化/嘶哑/吞咽困难等 | 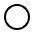 0 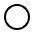 1 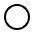 2 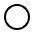 3  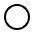 4 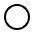 5 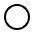 6 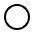 7 | 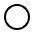 0 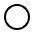 1 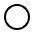 2 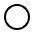 3  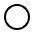 4 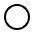 5 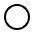 6 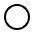 7 |  | 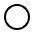 0 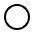 1 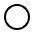 2 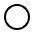 3  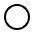 4 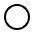 5 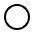 6 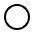 7 |
| 1.Throat sensitivity/voice changes/hoarseness/difficulty swallowing, etc. | 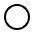 0 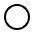 1 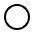 2 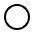 3  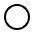 4 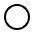 5 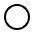 6 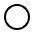 7 | 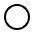 0 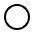 1 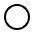 2 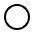 3  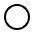 4 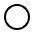 5 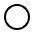 6 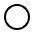 7 |  | 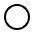 0 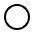 1 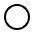 2 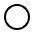 3  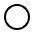 4 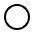 5 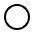 6 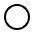 7 |
| 2.咳嗽/咳中带血 | 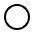 0 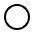 1 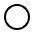 2 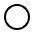 3  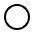 4 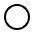 5 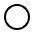 6 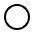 7 | 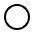 0 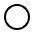 1 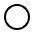 2 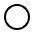 3  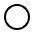 4 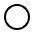 5 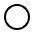 6 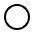 7 |  | 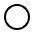 0 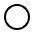 1 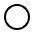 2 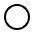 3  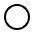 4 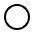 5 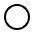 6 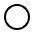 7 |
| 2.Coughing/coughing up blood | 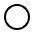 0 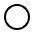 1 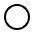 2 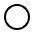 3  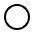 4 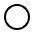 5 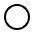 6 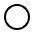 7 | 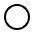 0 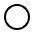 1 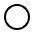 2 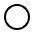 3  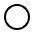 4 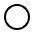 5 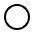 6 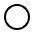 7 |  | 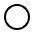 0 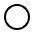 1 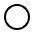 2 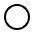 3  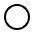 4 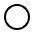 5 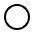 6 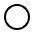 7 |
| 3.流鼻涕/鼻塞 | 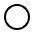 0 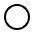 1 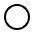 2 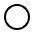 3  4  5  6  7 | 0  1  2  3  4  5  6  7 |  | 0  1  2  3  4  5  6  7 |
| 3.Runny/stuffy nose | 0  1  2  3  4  5  6  7 | 0  1  2  3  4  5  6  7 |  | 0  1  2  3  4  5  6  7 |
| 4.嗅觉/味觉减弱 | 0  1  2  3  4  5  6  7 | 0  1  2  3  4  5  6  7 |  | 0  1  2  3  4  5  6  7 |
| 4.Decreased sense of smell/taste | 0  1  2  3  4  5  6  7 | 0  1  2  3  4  5  6  7 |  | 0  1  2  3  4  5  6  7 |
| 5.食欲减少/体重减少 | 0  1  2  3  4  5  6  7 | 0  1  2  3  4  5  6  7 |  | 0  1  2  3  4  5  6  7 |
| 5.Loss of appetite/weight loss | 0  1  2  3  4  5  6  7 | 0  1  2  3  4  5  6  7 |  | 0  1  2  3  4  5  6  7 |
| 6.发热/发烧 | 0  1  2  3  4  5  6  7 | 0  1  2  3  4  5  6  7 |  | 0  1  2  3  4  5  6  7 |
| 6.Fever | 0  1  2  3  4  5  6  7 | 0  1  2  3  4  5  6  7 |  | 0  1  2  3  4  5  6  7 |
| 7.恶心/呕吐 | 0  1  2  3  4  5  6  7 | 0  1  2  3  4  5  6  7 |  | 0  1  2  3  4  5  6  7 |
| 7.Nausea/vomiting | 0  1  2  3  4  5  6  7 | 0  1  2  3  4  5  6  7 |  | 0  1  2  3  4  5  6  7 |
| 8.疲倦/困倦/无精打采 | 0  1  2  3  4  5  6  7 | 0  1  2  3  4  5  6  7 |  | 0  1  2  3  4  5  6  7 |
| 8.Fatigue | 0  1  2  3  4  5  6  7 | 0  1  2  3  4  5  6  7 |  | 0  1  2  3  4  5  6  7 |
| 9.浑身肌肉关节酸痛/头痛/咽喉痛/嗓子痛/眼睛痛等 | 0  1  2  3  4  5  6  7 | 0  1  2  3  4  5  6  7 |  | 0  1  2  3  4  5  6  7 |
| 9.Pain | 0  1  2  3  4  5  6  7 | 0  1  2  3  4  5  6  7 |  | 0  1  2  3  4  5  6  7 |
| 10.情绪波动（担心/焦虑/愤怒/生气/低落/抑郁等） | 0  1  2  3  4  5  6  7 | 0  1  2  3  4  5  6  7 |  | 0  1  2  3  4  5  6  7 |
| 10.Emotional swings (worry / anxiety / anger / anger / depression / depression etc.) | 0  1  2  3  4  5  6  7 | 0  1  2  3  4  5  6  7 |  | 0  1  2  3  4  5  6  7 |
| 11.认知（记忆/注意力/做计划）有困难 | 0  1  2  3  4  5  6  7 | 0  1  2  3  4  5  6  7 |  | 0  1  2  3  4  5  6  7 |
| 11.Difficulties with cognition (memory/concentration/planning) | 0  1  2  3  4  5  6  7 | 0  1  2  3  4  5  6  7 |  | 0  1  2  3  4  5  6  7 |
| 12.在体力劳动或脑力劳动后，感到症状加重 | 0  1  2  3  4  5  6  7 | 0  1  2  3  4  5  6  7 |  | 0  1  2  3  4  5  6  7 |
| 12.Symptoms that get worse after physical or mental exertion | 0  1  2  3  4  5  6  7 | 0  1  2  3  4  5  6  7 |  | 0  1  2  3  4  5  6  7 |
| 13.活动时感到心悸/气喘吁吁/心跳加速/头晕等 | 0  1  2  3  4  5  6  7 | 0  1  2  3  4  5  6  7 |  | 0  1  2  3  4  5  6  7 |
| 13.Feeling palpitations/shortness of breath/rapid heartbeat/dizziness during activities, etc. | 0  1  2  3  4  5  6  7 | 0  1  2  3  4  5  6  7 |  | 0  1  2  3  4  5  6  7 |
| 14.行动（洗澡或穿衣/做家务或购物/照顾家人等活动）有困难 | 0  1  2  3  4  5  6  7 | 0  1  2  3  4  5  6  7 |  | 0  1  2  3  4  5  6  7 |
| 14.Difficulty with mobility (such as bathing or dressing/housework or shopping/caring for family members) | 0  1  2  3  4  5  6  7 | 0  1  2  3  4  5  6  7 |  | 0  1  2  3  4  5  6  7 |
| 15.沟通交流受限 | 0  1  2  3  4  5  6  7 | 0  1  2  3  4  5  6  7 |  | 0  1  2  3  4  5  6  7 |
| 15.Limited communication | 0  1  2  3  4  5  6  7 | 0  1  2  3  4  5  6  7 |  | 0  1  2  3  4  5  6  7 |

Reference:

World Health Organization. The first few X cases and contacts (‎FFX)‎ investigation protocol for coronavirus disease 2019 (‎COVID-19). version 2.2. World Health Organization; 2020. Available from: https://apps.who.int/iris/handle/10665/332023
